# Integrated transcriptomics reveals the brain and blood biomarkers in Alzheimer's disease

**DOI:** 10.1111/cns.14316

**Published:** 2023-06-19

**Authors:** Haitao Yu, Fangzhou Wang, Jia‐jun Wu, Juan Gong, Shuguang Bi, Yumin Mao, Dongdong Jia, Gao‐shang Chai

**Affiliations:** ^1^ Department of Fundamental Medicine, Wuxi School of Medicine Jiangnan University Wuxi China; ^2^ The Affiliated Mental Health Center of Jiangnan University Wuxi Central Rehabilitation Hospital Wuxi China

**Keywords:** Alzheimer's disease, ATP6V1E1, blood, entorhinal cortex, peripheral biomarkers, transcriptomics

## Abstract

**Background:**

The systematic molecular associations between the peripheral blood cells and brain in Alzheimer's disease (AD) remains unclear, which hinders our understanding of AD pathological mechanisms and the exploration of new diagnostic biomarkers.

**Methods:**

Here, we performed an integrated analysis of the brain and peripheral blood cells transcriptomics to establish peripheral biomarkers of AD. By employing multiple statistical analyses plus machine learning, we identified and validated multiple regulated central and peripheral network in patients with AD.

**Results:**

By bioinformatics analysis, a total of 243 genes were differentially expressed in the central and peripheral systems, mainly enriched in three modules: immune response, glucose metabolism and lysosome. In addition, lysosome related gene ATP6V1E1 and immune response related genes (IL2RG, OSM, EVI2B TNFRSF1A, CXCR4, STAT5A) were significantly correlated with Aβ or Tau pathology. Finally, receiver operating characteristic (ROC) analysis revealed that ATP6V1E1 showed high‐diagnostic potential for AD.

**Conclusion:**

Taken together, our data identified the main pathological pathways in AD progression, particularly the systemic dysregulation of the immune response, and provided peripheral biomarkers for AD diagnosis.

## Graphical abstract

Here, we performed an integrated analysis of brain and peripheral blood cells transcriptomics to establish peripheral biomarkers of AD. By employing multiple statistical analyses plus machine learning, we identified and validated multiple regulated central and peripheral network in AD patients, which provided rich resources for understanding the pathogenesis of AD and large‐scale biomarker validation.

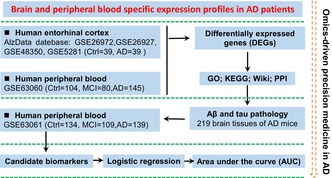

## 1 INTRODUCTION

Alzheimer's disease (AD) is the most common neurodegenerative disease, and its main clinical manifestations are cognitive impairment and progressive decline of memory ability.^1^ The main pathology of AD includes amyloid (Aβ) plaque deposition and neurofibrillary tangles (NFTs) formed by hyperphosphorylation Tau.^2^, ^3^ With the acceleration of population aging, the prevalence of AD has increased dramatically. In 2018, Alzheimer's Disease International (ADI) estimated that there were about 50 million people with dementia in worldwide, and it is expected that the global prevalence will increase by 2 times by 2050.^2^ Therefore, effective diagnosis is very important for the prevention and treatment of AD. Currently, the main diagnostic methods for AD include MRI and PET brain imaging, biochemical analysis of Aβ42/40, and total tau (t‐tau) and phosphorylated tau (p‐tau181) levels in the cerebrospinal fluid (CSF).^4^, ^5^, ^6^ The above methods are expensive and invasive, and are not easy to be accepted by the public. Therefore, convenient and noninvasive blood biomarkers for the diagnosis of AD are urgently needed.

Blood biomarkers are widely used in metabolic, immune, or cardiovascular diseases.^7^, ^8^, ^9^ Therefore, the study of AD blood biomarkers is a very promising direction. Previous studies have revealed the molecular alterations of patients with AD and identified a series of biomarkers, but most of them are limited to blood,^10^, ^11^ and the relevant peripheral and central integration is still missing. The entorhinal cortex (EC), thought to be the location of the earliest lesions in AD, and the degree of atrophy in EC reflects the early pathological changes of AD and can be a strong predictor of prodromal AD.^12^ Therefore, the integration of EC and peripheral blood cells transcriptomics is an interesting direction, providing a window to understand the altered pathological mechanisms of AD brain from the blood, which is crucial for exploring reliable peripheral diagnostic biomarkers.

Here, we integrated transcriptomic data from the entorhinal cortex (EC) and peripheral blood cells to reflect the central and peripheral connections in patients with AD. Immune response, glucose metabolism, and lysosomes may be closely related to the progression of AD. Lysosome‐associated genes ATP6V1E1 and immune response‐related genes (IL2RG, OSM, EVI2B TNFRSF1A, CXCR4, STAT5A) were significantly correlated with Aβ or Tau pathology, which may be candidate targets for AD treatment. Receiver operating characteristic (ROC) analysis revealed that ATP6V1E1 showed high‐diagnostic potential for AD.

## 2 MATERIALS AND METHODS

### 2.1 Data acquisition

All the datasets used for the exploration of brain and peripheral specific expression profiles in mild cognitive impairment (MCI) and patients with AD are from the National Biotechnology Information Center (NCBI) Gene Expression Comprehensive Database (GEO).^13^ Specifically, EC transcriptome data were obtained from GSE26972, GSE26927, GSE48350, GSE5281, which have been integrated in the Alzdata database (http://www.alzdata.org/),^14^ for a total of 39 AD patients and 39 normal aging subjects. The Alzdata database has integrated and normalized multiple datasets across platforms, providing a reliable data resource for the exploration of pathological mechanisms and drug development related to AD. Peripheral blood cells transcriptomes were obtained from GSE63060 (Ctrl = 104, MCI = 80, AD = 145) and GSE63061 (Ctrl = 134, MCI = 109, AD = 139), which were two large European blood cell RNA expression datasets, and contributing to find reliable peripheral biomarkers for AD diagnosis.

### 2.2 Enrichment analysis and protein–protein interaction (PPI)

Genes with *p* < 0.05, |log2 Fold change (FC)| > 0.1375 (EC brain region), |log Fold change (FC)| > 0.0415 (peripheral blood cells) were defined as differentially expressed. To show the apparently altered pathophysiological processes in AD, WEB‐based GEne SeT AnaLysis Toolkit (http://www.webgestalt.org) were performed for biological process (BP), Kyoto Encyclopedia of genes and genomes (KEGG), and Wiki pathway enrichment analyses with differentially expressed genes (DEGs). Cytoscape 3.8.2 and STRING (v11; https://string‐db.org/) plug‐in were used for visual analysis of protein–protein interaction (PPI) network, and Molecular Complex Detection (MCODE) analysis was performed to seek the core DEGs set.

### 2.3 Correlation of potential genes with Aβ and tau pathology in AD mouse models

To study the correlation between module‐related genes and Aβ or Tau pathology, we examined the potential genes mRNA expression and the corresponding Aβ and Tau pathological grading scores in the 2AD mouse models brain tissues (www.mouseac.org).^15^ Specifically, immunofluorescence was applied to perform Aβ_40_ and phosphorylated Tau semi‐quantitative analysis, of which 165 brain tissue samples with Aβ pathological grading from four AD transgenic mice with human APP, PSEN1 mutant (TAS10 [with APP^K670N/M671L^ mutant], TPM [with PSEN1^M146V^ mutant], HO_ TASTPM [with homozygous mutant of APP and PSEN1 mentioned above], HET_TASTPM [with heterozygous mutant of APP and PSEN1 mentioned above]), and 42 brain tissue samples with Tau pathological grading from TAU transgenic mice (MAPT^P301L^ mutant).^15^ Graphpad prism software (graphpad software, La Jolla, CA, USA) was used for Pearson correlation analysis between gene mRNA expression and Aβ or Tau pathology scores.

### 2.4 Statistical analysis

Logistic regression analysis and ROC curves with area under the curve (AUC) were used to evaluate the efficiency of candidate biomarkers to discriminate between AD and normal subjects. All the data have been tested for normality with the D'Agostino & Pearson test. Non normally distributed data were analyzed by the non‐parametric equivalent. The Student's *t*‐test was used to evaluate the level of significance between the two groups. The data were expressed as mean ± SEM and *p* values <0.05 was considered to be significant.

## 3 RESULTS

### 3.1 Study design and rationale

**FIGURE 1:**
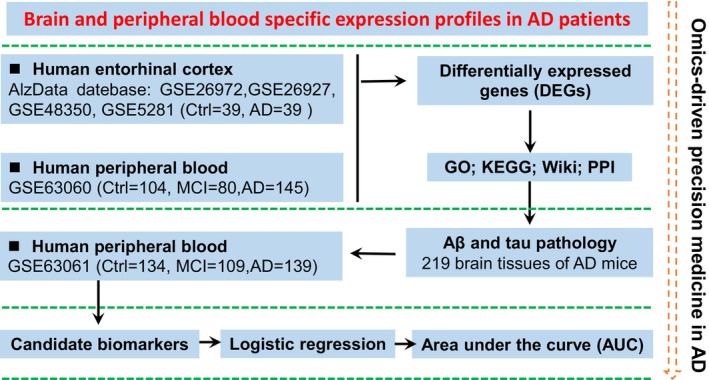
Rationale and workflow of the present study. A systematic transformation pipeline, including bioinformatics analyses, such as Veen analysis, KEGG, Wiki pathway, GO, and PPI, was constructed to integrate the central and peripheral multiple transcriptomics data. Using this platform, the central‐peripheral linkage modules and candidate diagnostic biomarkers were identified. AUC, Area under the curve; GO, Gene Ontology; KEGG, Kyoto Encyclopedia of Genes and Genomes; PPI, protein–protein interaction network.

The research flowchart is shown in Figure 1. At first, the integrated EC and peripheral blood cells transcriptome data were used to reveal genes that were commonly altered centrally and peripherally of patients with AD. Then, bioinformatics analysis was used to reflect the rich pathophysiological mechanisms covered by the DEGs. Further, correlation analysis between potential genes with Aβ and Tau pathology was performed for screening candidate biomarkers. Finally, logistic regression and ROC analysis were used to reveal the diagnostic efficiency of candidate molecules. The whole framework demonstrates the application of omics‐driven precision medicine in AD.

### 3.2 Common differentially expressed genes and enrichment analysis of EC and blood in patients with AD

**FIGURE 2:**
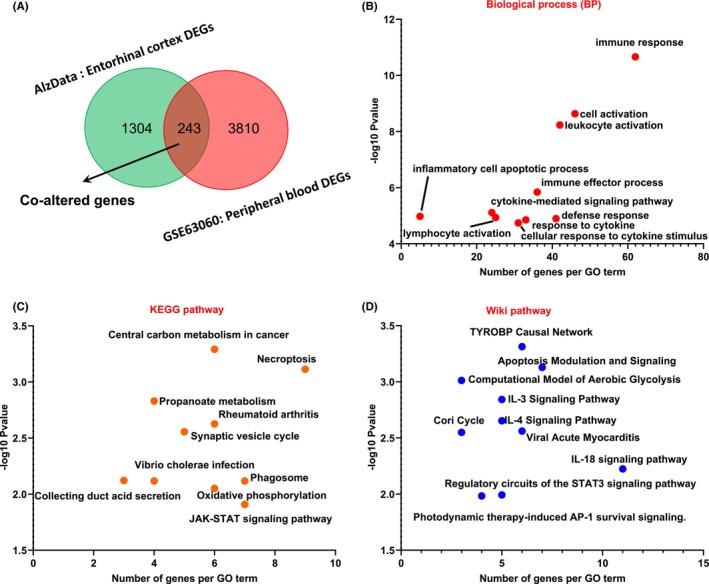
Common differentially expressed genes and enrichment analysis of entorhinal cortex and blood in patients with AD. (A) Venn diagram showing the 243 coaltered genes in entorhinal cortex and blood in patients with AD. (B–D) Coaltered genes are enriched in different biological processes (BP), KEGG, and wiki pathway. The −log10 (*p*) was used to define the enrichment strength.

A total of 1547 DEGs were found in EC brain regions and 4053 DEGs were found in peripheral blood cells, of which 243 DEGs were coaltered (Figure 2A; Appendix S1). Biological process enrichment analysis showed that coaltered genes were mainly enriched in immune response, cell activation, leukocyte activation, immune effector process, lymphocyte activation, inflammatory cell apoptotic process, cytokine‐mediated, signaling pathway, defense response, response to cytokine, cellular response to cytokine stimulus (Figure 2B). The KEGG pathway enrichment analysis showed that coaltered genes were mainly enriched in central carbon metabolism in cancer, necroptosis, propanoate metabolism, rheumatoid arthritis, synaptic vesicle cycle, collecting duct acid secretion, vibrio cholerae infection, phagosome, oxidative phosphorylation, JAK–STAT signaling pathway (Figure 2C). Wiki pathway enrichment analysis showed that coaltered genes were mainly enriched in TYROBP causal network, apoptosis modulation and signaling, computational model of aerobic glycolysis, IL‐3 signaling pathway, IL‐4 signaling pathway, viral acute myocarditis, cori cycle, IL‐18 signaling pathway, regulatory circuits of the STAT3 signaling pathway, photodynamic therapy‐induced AP‐1 survival signaling (Figure 2D). Together, patients with AD showed complex pathological changes, especially systemic central and peripheral immune response.

### 3.3 The central and peripheral linkage MCODE based on PPI network analysis

**FIGURE 3:**
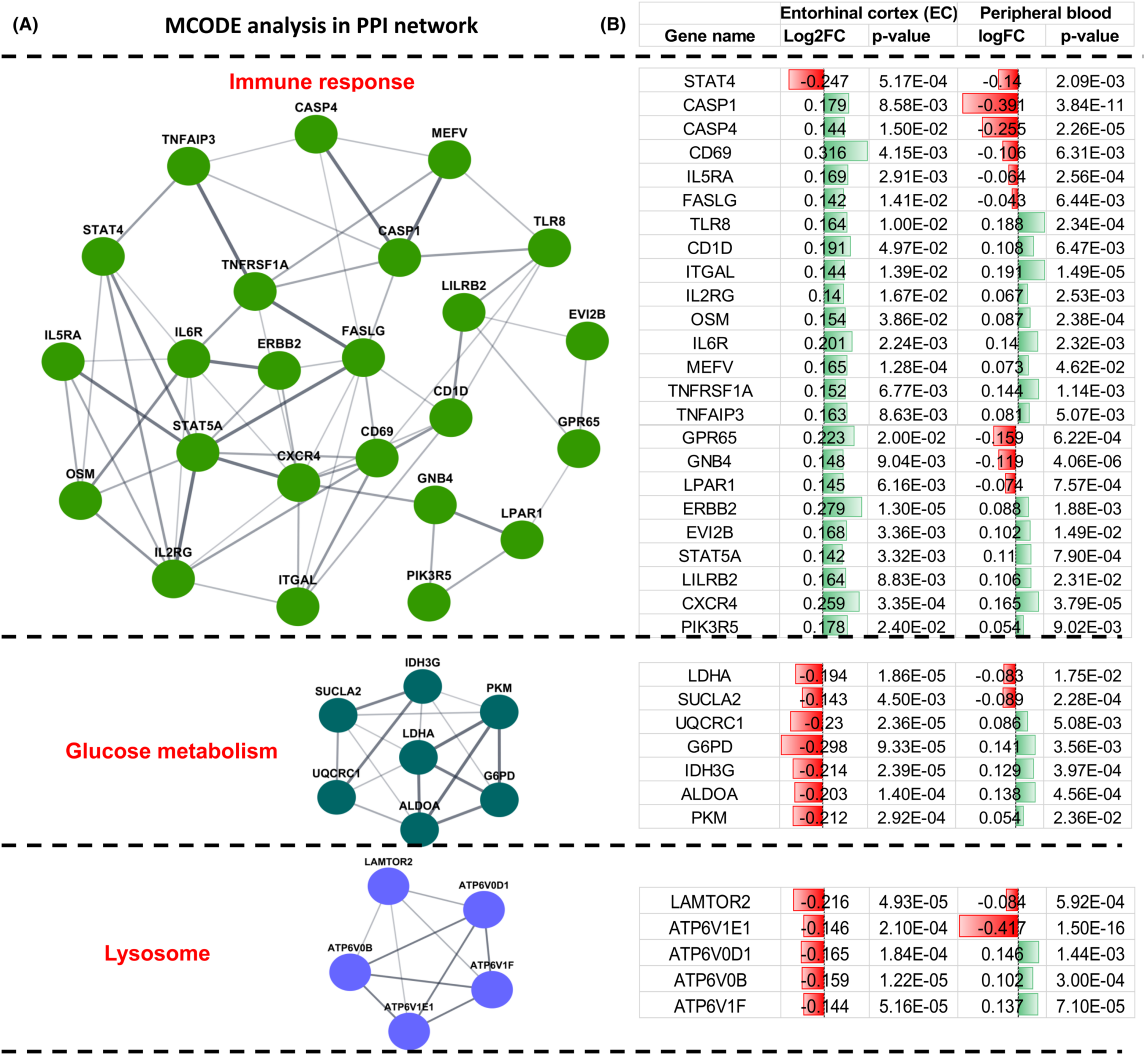
The central and peripheral linkage MCODE based on PPI network analysis. The core modules and functions enriched by coaltered genes, as well as specific molecules and fold changes. MCODE, Molecular Complex Detection; PPI, Protein–protein interaction.

To further identify key modules and related molecules, we conducted Molecular Complex Detection (MCODE) analysis based on PPI networks. Modules analysis showed that the coaltered genes were mainly related to immune response, glucose metabolism, and lysosomes (Figure 3). Interestingly, genes involved in immune response showed overall up‐regulation, while genes involved in glucose metabolism and lysosomes showed overall downregulation in the brain (Figure 3). However, 16 MCODE‐related genes showed an opposite trend in the peripheral system, which may be related to the complexity of the blood cell system and be more susceptible to other diseases or environmental influences (Figure 3). To find more objective peripheral diagnostic biomarkers to reflect pathological changes in the brain, 20 MCODE‐related genes with the consistent change were selected for subsequent analysis.

### 3.4 Correlation of MCODE‐related genes with Aβ and tau pathology in AD mouse models

**FIGURE 4:**
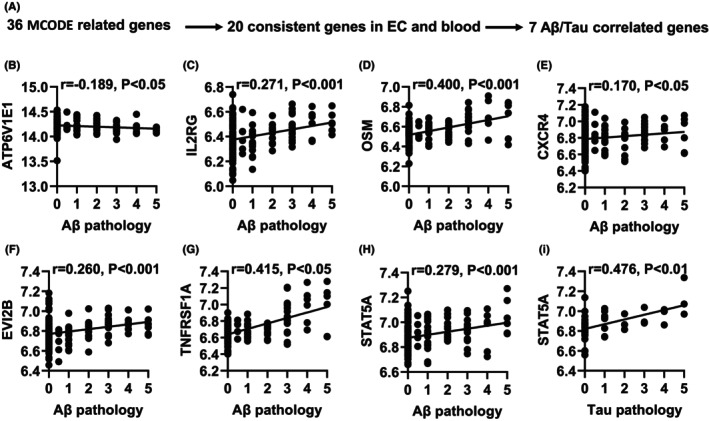
Correlation of MCODE related genes with Aβ and tau pathology in AD mouse models. (A) Correlation analysis gene determination process (B–I) Pearson correlation coefficients (*r*) and corresponding *p* values <0.05 were displayed at the top of each plot. X‐axis shows Aβ or Tau pathology score, y‐axis indicates relative expression abundance of each gene.

To determine the association between MCODE‐related genes and AD progression, we analyzed the expression of MCODE‐related genes in AD mice and correlated them with Aβ and Tau pathology (Figure 4A). V‐type proton ATPase subunit E 1 (ATP6V1E1), a subunit of the V1 complex of vacuolar (H+)‐ATPase (V‐ATPase), was involved in the maintenance of lysosomal acidic environment, which may participate in the AD development by affecting the degradation of Aβ.^16^ Pearson correlation analysis found that the ATP6V1E1 expression showed a significant negative correlation with Aβ pathology (*r* = −0.189, *p* < 0.05; Figure 4B), suggesting that its decrease may be related to Aβ deposition. Immune dysfunction was the main feature of AD, and peripheral‐central immune crosstalk was closely related to AD pathology.^17^ In this study, we found that immune‐response–related genes (IL2RG, OSM, EVI2B TNFRSF1A, CXCR4, STAT5A) showed a significant positive correlation with Aβ and Tau (*r* = 0.170–0.415, *p* < 0.05; Figure 4C–I), suggesting that their increase may activate the immune response to exacerbate AD pathology.

### 3.5 Expression level and diagnostic efficiency of candidate biomarkers

**FIGURE 5:**
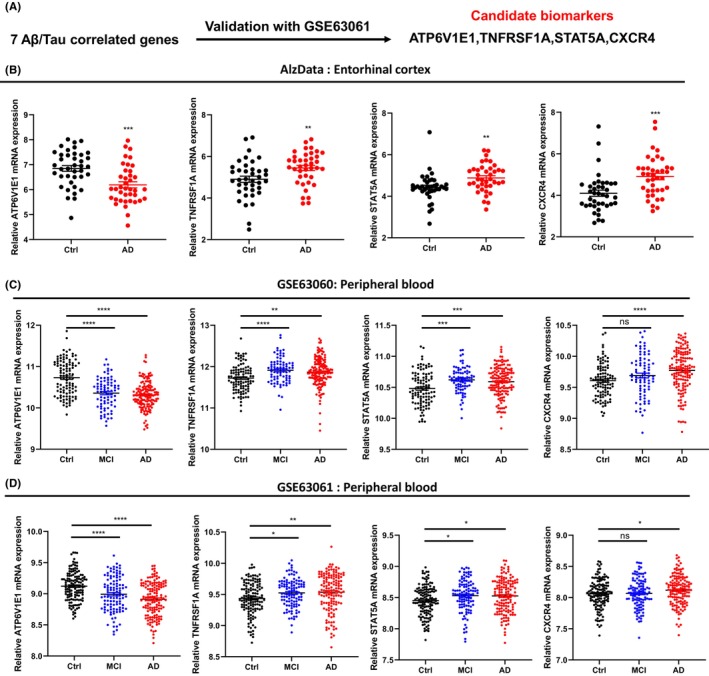
Expression level of candidate biomarkers in entorhinal cortex and peripheral blood of patients withg AD. (A) Candidate biomarkers identified through GSE63061 dataset validation. (B–D) Expression level of candidate biomarkers in entorhinal cortex and peripheral blood of patients with AD. Data were shown as mean ± SEM. *p < 0.05, **p < 0.01, ***p < 0.001, and ****p < 0.0001.

To investigate whether the Aβ/Tau correlated genes have diagnostic potential, we analyzed their expression levels in the brain and peripheral blood cells (Figure 5A–D). With the GSE63061 dataset validation, we found four Aβ/Tau correlated genes (ATP6V1E1, TNFRSF1A, CXCR4, STAT5A) were also significantly different in MCI and AD patients (Figure 5A). Specifically, lysosome‐related gene ATP6V1E1 was significantly decreased in brain tissue and peripheral blood cells, while immune response‐related genes TNFRSF1A, CXCR4, STAT5A were significantly increased (Figure 5B–D).

**FIGURE 6:**
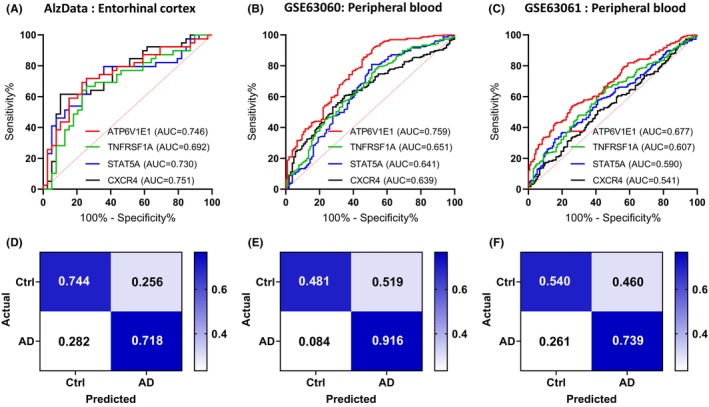
Efficiency of candidate biomarkers for the AD diagnosis. (A–C) ROC curve for the candidate biomarkers of AD based on based on AlzData (A), GSE63060 (B), and GSE63061 (C). (D–F) The confusion matrix of the candidate biomarker ATP6V1E1 based on AlzData (D), GSE63060 (E), and GSE63061 (F).

To analyze the performance of candidate biomarkers in brain and the peripheral blood cells to distinguish patients with AD or MCI from controls, we performed a ROC analysis. ROC analysis found that ATP6V1E1 showed good discrimination in both brain and peripheral blood cells (EC region: AUC = 0.746, *p* < 0.001, Specificity = 0.744, Sensitivity = 0.718; GSE63060: AUC = 0.759, *p* < 0.001, Specificity = 0.481, Sensitivity = 0.916; GSE63061: AUC = 0.677, *p* < 0.001, Specificity = 0.540, Sensitivity = 0.739; Figure 6A–F). This study demonstrates the application of omics driven precision medicine in AD, which may contribute to the development of more convenient clinical detection methods in the future.

## 4 DISCUSSION

Alzheimer's disease (AD) is the most common neurodegenerative disease, and with the acceleration of population aging, the prevalence of AD has increased dramatically. Therefore, the convenient and non‐invasive peripheral biomarkers for AD early diagnosis is an urgent need. In this study, we focused on the transcriptome data of the EC and peripheral blood mononuclear cells (PBMC), as the EC brain region is the earliest lesion area of AD,^12^ and the transcriptome data of blood cells can best reflect the alterations in the peripheral system. By integrating the transcriptome data of brain regions and PBMCs, 243 differentially expressed genes were coaltered, which were significantly enriched in immune response, glucose metabolism and lysosome‐related modules. Furthermore, the correlation analysis of mRNA expression with Aβ and Tau pathology identified 7 candidate biomarkers that may be involved in the development of AD. The resulting candidate biomarkers have the potential to distinguish AD from normal subjects.

The cross‐talk between glial cells in the central system and peripheral immune cells invading the brain is closely related to the progression of AD,^18^ especially in the early stages, when the peripheral immune cells would induce glial activation, pro‐inflammatory cytokine release and neuronal damage.^19^, ^20^ Further, neuroinflammation can induce the formation of Aβ amyloid deposition and Tau pathology.^19^ We found that central and peripheral coaltered genes were significantly enriched in immune‐response–related processes, suggesting that associated inflammation may be a major contributor to AD pathology. Recent studies have shown that the T cells can activate microglia, thereby promoting the development of Tau pathology in neurodegenerative diseases including AD, while blocking or depleting T cells can significantly improve the learning and memory function and related pathology in AD mouse models.^21^ Furthermore, neutrophils mediate inflammatory effects in the 3xTg‐AD mice brain via lymphocyte function‐associated antigen 1 (LFA‐1) integrin, thereby damaging neurons and accelerating cognitive impairment.^22^ Moreover, immune‐response–related genes (TNFRSF1A, CXCR4, STAT5A) showed a significant positive correlation with Aβ and Tau, suggesting that their increase may activate the immune response to exacerbate AD pathology. Tumor negative factor acceptor superfamily member 1A (TNFRSF1A), as an acceptor for TNFSF2/TNF‐alpha and homologous TNFSF1/lipoxin‐alpha, can mediate neuroinflammation and neuronal excitotoxicity,^23^ and is a high‐risk gene for AD.^24^ C‐X‐C chemokine receptor type 4 (CXCR4) synergizes with the chemokine CXCL12 to mediate inflammatory responses and cognitive decline in patients with AD.^25^ Signal transducer and activator of transcription 5A (STAT5A) is involved in JAK2–STAT5A signaling pathway and then mediates IL‐3‐activated microglia.^26^ Together, different types of immune cell infiltration involve complex signal transduction, which is worth further exploration in future diagnosis and treatment.

In the early stage of AD, glucose utilization in the cortex gradually declines, and the degree is closely related to the AD process,^27^ cerebral blood flow was impaired by 20%–65% and cerebral glucose utilization was reduced by nearly 45% during AD.^28^ Decreased glucose metabolism can lead to energy deficiency, mitochondrial damage, and then induce neuroinflammation to accelerate AD pathology.^29^ Moreover, synaptic transmission requires a large amount of ATP, so mitochondrial damage can lead to synaptic dysfunction.^30^ Previously, proteomic studies have shown that mitochondria‐related proteins were significantly reduced in the plasma of AD patients, so glucose metabolism disorder may be a systemic disorder in AD patients.^11^ Glucose metabolism involves multiple processes including glycolysis, tricarboxylic acid cycle (TCA), and oxidative phosphorylation.^31^ Here, we found that enzymes related to glycolysis, TCA, and oxidative phosphorylation were significantly decreased in AD brain. In addition, L‐lactate dehydrogenase A chain (LDHA) and succinate–CoA ligase [ADP‐forming] subunit beta (SUCLA2) were also significantly decreased in blood cells. LDHA was involved in catalyzing the conversion of lactate and NAD to pyruvate and NADH,^32^ and its overexpression can slow down Aβ‐induced neurotoxicity by reducing ROS production and enhancing glycolysis.^33^ SUCLA2 was a key rate‐limiting enzyme in the TCA cycle, which can promote the phosphorylation of TCA substrates, and its reduction leads to ATP deficiency and aerobic metabolism disorder.^34^ There may be a disconnect between the changes of central and peripheral glucose metabolism‐related genes, which may be due to the blood–brain barrier (BBB), and the environment in the brain is simpler, while the peripheral system may be affected by age‐related underlying diseases, such as diabetes, hypertension, and hyperlipidemia, etc. Therefore, the consistent downregulation of glucose metabolism‐related genes in the brain may explain the gradual decrease of glucose utilization in patients with AD.

Lysosomes are widespread within eukaryotic cells, which mediate the degradation of metabolic waste, toxic proteins, and damaged organelles enclosed by autophagosomes and endosomes,^35^ and play an important role in programmed cell death, plasma membrane repair, development, and cell differentiation.^36^ Lysosomal dysfunction caused by age or other factors lead to decreased lysosomal proteolytic activity and accumulation of damaged organelles or toxic proteins, leading to a variety of neurodegenerative diseases including AD and Parkinson's disease (PD).^36^ The normal functioning of the lysosome is closely linked to lysosomal pH, and decreased lysosomal acidity causes Aβ aggregation, exacerbating neuronal damage.^16^ Here, we found that lysosomal channel proteins (ATP6V0B, ATP6V1E1, ATP6V0D1, ATP6V1F) were significantly decreased in AD, which may mediate the elevation of lysosomal pH leading to lysosomal disorders. In addition, correlation analysis showed that ATP6V1E1 was significantly negatively correlated with Aβ, suggesting that its decrease may have promoted Aβ aggregation. The diagnostic analysis showed that ATP6V1E1 had a high potential to distinguish AD from normal subjects. Together, ATP6V1E1 may be an important target for the diagnosis and treatment of AD. Interestingly, four genes (ATP6V0B, ATP6V1E1, ATP6V0D1, ATP6V1F) are subunits of vacuolar ATPase (V‐ATPase), which hydrolyzes ATP to release energy and transports protons,^37^ and can also participate in glucose metabolic response through oxidative phosphorylation.

## 5 CONCLUSIONS

In sum, we have integrated the molecular connections between brain and blood cells through a systems biology approach, providing a valuable data resource for the early diagnosis and treatment of AD. Bioinformatics analysis suggested that we should focus on immune response, glucose metabolism, as well as lysosome dysfunctions in future studies of AD. The lysosomal ion channel protein ATP6V1E1 may be an ideal candidate biomarker for AD diagnosis. Overall, the exploration of integrating central and peripheral transcriptomics reflects the application of omics‐driven precision medicine in AD.

## AUTHOR CONTRIBUTIONS

*Experimental design*: Haitao Yu, Dongdong Jia and Gao‐shang Chai. *Experimental methods*: Haitao Yu, Dongdong Jia, Jia‐jun Wu and Gao‐shang Chai. *Data analysis*: Haitao Yu, Fangzhou Wang, Jia‐jun Wu, Juan Gong, Yumin Mao and Shuguang Bi. *Manuscript‐writing*: Haitao Yu, Dongdong Jia and Gao‐shang Chai.

## CONFLICT OF INTEREST STATEMENT

The authors declare that they have no conflict of interest to disclose.

## Supporting information


Appendix S1.

## Data Availability

All data used to support the findings of this study are included within the article. Gene expression profiles of large samples of EC can be obtained from the AlzData database (http://www.alzdata.org/). Transcriptomics of peripheral blood cells can be found here: https://www.ncbi.nlm.nih.gov/geo/query/acc.cgi?acc=GSE63060; https://www.ncbi.nlm.nih.gov/geo/query/acc.cgi?acc=GSE63061. The mRNA expression profile, Aβ and Tau pathological scores in AD mouse models (www.mouseac.org).
